# Approximation degree of Durrmeyer–Bézier type operators

**DOI:** 10.1186/s13660-018-1622-1

**Published:** 2018-02-22

**Authors:** Purshottam N. Agrawal, Serkan Araci, Martin Bohner, Kumari Lipi

**Affiliations:** 1 Department of Mathematics, Indian Institute of Technology Roorkee, Roorkee, India; 2 Department of Economics, Faculty of Economics, Administrative and Social Sciences, Hasan Kalyoncu University, Gaziantep, Turkey; 3 Department of Mathematics and Statistics, Missouri University of Science and Technology, Rolla, USA

**Keywords:** 26A15, 40A35, 41A25, 41A36, Baskakov–Szász type operators, Rate of convergence, Bounded variation, Ditzian–Totik modulus of smoothness

## Abstract

Recently, a mixed hybrid operator, generalizing the well-known Phillips operators and Baskakov–Szász type operators, was introduced. In this paper, we study Bézier variant of these new operators. We investigate the degree of approximation of these operators by means of the Lipschitz class function, the modulus of continuity, and a weighted space. We study a direct approximation theorem by means of the unified Ditzian–Totik modulus of smoothness. Furthermore, the rate of convergence for functions having derivatives of bounded variation is discussed.

## Introduction

For a continuous function *h* on $[0,1]$, Bernstein [1] defined a linear positive operator in order to provide a very simple and elegant proof of the Weierstrass approximation theorem, namely

$$B_{n}(h;x)=\sum_{k=0}^{n} \binom{n}{k}x^{k}(1-x)^{n-k} h \biggl( \frac{k}{n} \biggr),\quad x\in[0,1]. $$

In order to approximate continuous functions on $[0,\infty)$, Szász [2] introduced the operator

1.1 $$ S_{n}(h;x)=\sum_{k=0}^{\infty} \frac{(nx)^{k}}{k!}e^{-nx} h \biggl( \frac{k}{n} \biggr), $$

provided the infinite series on the right-hand side converges. Later on, for $h\in \operatorname {C}[0,\infty)$ and $0\leq\beta<1$, Jain [3] proposed a modification of the operators given in (1.1), namely

1.2 $$ P_{n}^{(\beta)}(h;x)=\sum _{k=0}^{\infty}L_{n,k}^{(\beta)}(x) h \biggl( \frac{k}{n} \biggr), $$

where

$$L_{n,k}^{(\beta)}(x)=\frac{nx(nx+k\beta)^{k-1}}{k!}e^{-(nx+k\beta)} $$

with the partition of unity

$$\sum_{k=0}^{\infty}L_{n,k}^{(\beta)}(x)=1. $$

It is observed that the Jain operator (1.2) includes the Szász operator (1.1) as a special case for $\beta=0$. Recently, Gupta and Greubel [4] also proposed the Durrmeyer type modification of the operators given in (1.2) as

$$D_{n}^{(\beta)}(h;x)=\sum_{k=0}^{\infty} \frac{\langle L_{n,k}^{( \beta)},h\rangle}{\langle L_{n,k}^{(\beta)},1\rangle} L_{n,k}^{( \beta)}(x), $$

where

$$\langle h,g\rangle= \int_{0}^{\infty}h(t)g(t)\,\mathrm {d}t. $$

They showed that these operators converge to *h* without any restriction on *β*. The moments for these operators were obtained by using Tricomi’s hypergeometric functions and Stirling numbers of first kind, and some approximation properties of these operators were proved.

In the literature, many authors have discussed the approximation behavior of different summation-integral type operators (see [5, 6]). For $0\leq\beta<1$ and $c\geq0$, Acu and Gupta [7] introduced mixed Durrmeyer type operators for $x\in[0, \infty)$ as

1.3 $$ P_{n}^{\beta,c}(h;x)=\sum _{k=1}^{\infty} \biggl( \int_{0}^{\infty}L _{n,k-1}^{[\beta]}(t)\,\mathrm {d}t\biggr) ^{-1}p_{n,k}(x,c) \int_{0}^{\infty}L _{n,k-1}^{[\beta]}(t)h(t) \,\mathrm {d}t+p_{n,0}(x,c)h(0), $$

where

$$p_{n,k}(x,c)=\frac{(-x)^{k}}{k!}\phi_{n,c}^{(k)}(x) $$

and

$$\phi_{n,c}(x)= \textstyle\begin{cases} e^{-nx}, & \text{if } c=0, \\ (1+cx)^{-n/c}, & \text{if } c>0. \end{cases} $$

They determined the degree of approximation by means of the modulus of continuity and a weighted space. The authors also studied the approximation of functions having derivatives equivalent with a function of bounded variation. It is observed that the operator defined by (1.3) has two special cases:

1. If $\phi_{n,c}(x)= e^{-nx}$ and $\beta=0$, then the Phillips operators are obtained [8].
2. If $\phi_{n,c}(x)=(1+cx)^{-n/c}$ and $\beta=0$, then one gets the Baskakov–Szász operators [9].

Zeng and Piriou [10] initiated the study of Bézier variant of Bernstein operators. Zeng and Chen [11] estimated the rate of approximation for Bézier–Bernstein–Durrmeyer operators. Zeng and Tao [12] considered Bézier–Baskakov–Durrmeyer operators for $\alpha\geq1$ and obtained the rate of convergence. For some other contributions in this direction, we refer to [13–21]. Motivated by the above research, we introduce the Bézier variant of the operator (1.3) as

1.4 $$\begin{aligned} P_{n,\alpha}^{\beta,c}(h;x) =& \sum_{k=1}^{\infty} \biggl( \int_{0} ^{\infty}L_{n,k-1}^{[\beta]}(t)\,\mathrm {d}t\biggr) ^{-1} R_{n,k}^{(\alpha)}(x,c) \int_{0}^{\infty}L_{n,k-1}^{[\beta]}(t)h(t) \,\mathrm {d}t+R_{n,0}^{(\alpha)}(x,c)h(0) \\ =& \int_{0}^{\infty}K_{n,\alpha }^{\beta,c}(x;t)h(t) \,\mathrm {d}t, \end{aligned}$$

where $\alpha\geq1$,

$$R_{n,k}^{(\alpha)}(x,c) = \bigl[ I_{n,k}(x,c) \bigr] ^{\alpha}- \bigl[ I _{n,k+1}(x,c) \bigr] ^{\alpha} \quad \text{with } I_{n,k}(x,c)=\sum_{j=k}^{ \infty}p_{n,j}(x,c), $$

and

$$K_{n,\alpha}^{\beta,c}(x;t) =\sum_{k=1}^{\infty} \biggl( \int_{0} ^{\infty}L_{n,k-1}^{[\beta]}(t)\,\mathrm {d}t\biggr) ^{-1} R_{n,k}^{(\alpha)}(x,c)L _{n,k-1}^{[\beta]}(t) +R_{n,0}^{(\alpha)}(x,c)\delta(t), $$

*δ* being the Dirac delta function. For $\alpha=1$, we note that $P_{n,\alpha}^{\beta,c}(h;x)=P_{n}^{\beta,c}(h;x)$.

Recently, Acar et al. [22] considered the Bézier variant of Bernstein–Durrmeyer type operators and studied the degree of approximation of functions having derivative of bounded variation. The order of approximation of summation-integral type operators for functions with derivatives of bounded variation is estimated in [13, 23–27].

The aim of this paper is to investigate the weighted approximation properties and a direct approximation result by means of the Ditzian–Totik modulus of smoothness $\omega_{\phi^{\tau}}(h;t)$, $0\leq\tau\leq1$, and the rate of convergence for functions having a derivative of bounded variation for the operators given by (1.4). Throughout this paper, *C* denotes a constant which may be different at each occurrence.

## Preliminaries

In the sequel, the following auxiliary results are used to prove the main results of the paper.

### Lemma 1

(see [7])

*For the* *mth order moment* $P_{n}^{\beta,c}(t^{m};x)$, $m=0,1,2$, *we obtain*

1. $P_{n}^{\beta,c}(1;x)=1$;
2. $P_{n}^{\beta,c}(t;x)=(1-\beta)x +\frac{\beta(2-\beta)}{n(1- \beta)}(1-\phi_{n,c}(x))$;
3. $P_{n}^{\beta,c}(t^{2};x)=(1-\beta)^{2} [ x^{2}+ \frac{x(1+cx)}{n} ] +\frac{(1+4\beta-2\beta^{2})}{n}x +\frac{ \beta^{2}(3-\beta)}{n^{2}(1-\beta)}(1-\phi_{n,c}(x))$.

*Consequently*, *for the* *rth order central moment* $\mu_{n,r}^{\beta,c}(x)=P _{n}^{\beta,c}((t-x)^{r};x)$, $r=0,1,2$, *one has*

1. $\mu_{n,0}^{\beta,c}(x)=1$;
2. $\mu_{n,1}^{\beta,c}(x) =-\beta x+\frac{\beta(2-\beta)}{n(1-\beta )}(1-\phi_{n,c}(x))$;
3. $\mu_{n,2}^{\beta,c}(x)= [ \beta^{2}+\frac{c(1-\beta)^{2}}{n} ] x ^{2} +\frac{2-4\beta-\beta^{2}+\beta^{3}+2\beta(2-\beta)\phi _{n,c}(x)}{n(1- \beta)}x +\frac{\beta^{2}(3-\beta)(1-\phi_{n,c}(x))}{n^{2}(1-\beta)}$.

### Lemma 2

(see [7])

*If* $\beta=\beta(n)\to0$ *as* $n\to\infty$ *and* $\lim_{n\to\infty}n \beta(n)=l\in {\mathbb {R}}$, *then*

1. $\lim_{n\to\infty}n\mu_{n,1}^{\beta,c}(x)=-lx$;
2. $\lim_{n\to\infty}n\mu_{n,2}^{\beta,c}(x)=x(cx+2)$;
3. $\lim_{n\to\infty}n^{2}\mu_{n,4}^{\beta,c}(x)=3x^{2}(cx+2)^{2}$.

### Remark 1

It is observed that

2.1 $$ P_{n,\alpha}^{\beta,c}(1;x) =\sum _{k=0}^{\infty}R_{n,k}^{(\alpha)}(x,c) = \bigl[ I_{n,0}(x,c) \bigr] ^{\alpha} = \Biggl[ \sum _{j=0}^{\infty}p_{n,k}(x,c) \Biggr] ^{\alpha}=1, $$

since $\sum_{j=0}^{\infty}p_{n,k}(x,c)=1$.

Let $\operatorname {C}_{\mathrm{B}}[0,\infty)$ denote the space of all continuous and bounded functions on $[0,\infty)$, where the norm is defined by

$$\Vert h\Vert =\sup_{[0,\infty)}\bigl\vert h(x)\bigr\vert . $$

### Lemma 3

*For every* $h\in \operatorname {C}_{\mathrm{B}}[0,\infty)$, *we have*

$$\bigl\Vert P_{n,\alpha}^{\beta,c}(h;\cdot)\bigr\Vert \leq \Vert h \Vert . $$

Lemma 3 can easily be proved using (2.1).

### Remark 2

We observe that

$$\begin{aligned} 0< R_{n,k}^{(\alpha)}(x,c) =& \bigl[ I_{n,k}(x,c) \bigr] ^{\alpha} - \bigl[ I_{n,k+1}(x,c) \bigr] ^{\alpha} \\ \leq&\alpha \bigl(I_{n,k}(x,c)-I_{n,k+1}(x,c)\bigr) \\ =& \alpha p_{n,k}(x,c), \end{aligned}$$

in view of the inequality

$$\bigl\vert a^{\alpha}-b^{\alpha}\bigr\vert \leq\alpha\vert a-b \vert\quad \text{for } 0 \leq a,b\leq1, \alpha\geq1. $$

Hence, from (1.4), we get

$$\bigl\vert P_{n,\alpha}^{\beta,c}(h;x)\bigr\vert \leq\alpha P_{n}^{\beta,c}\bigl(\vert h\vert;x\bigr). $$

## Main results

For $x\in(0,\infty)$, $t\in[0,\infty)$, and $0< r\leq1$, as we can see in Özarslan and Duman [28], the Lipschitz type space is defined as

$$\operatorname {Lip}_{M}^{*}(r):= \biggl\{ h\in \operatorname {C}[0,\infty): \bigl\vert h(t)-h(x)\bigr\vert \leq M \frac{\vert t-x\vert ^{r}}{(t+x)^{r/2}} \biggr\} . $$

In the following theorem, we obtain the rate of convergence of the operators $P_{n,\alpha}^{\beta,c}$ for functions in $\operatorname {Lip}_{M}^{*}(r)$.

### Theorem 1

*Let* $h\in \operatorname {Lip}_{M}^{*}(r)$ *and* $r\in(0,1]$. *Then*, *for all* $x\in(0,\infty)$, *we have*

$$\bigl\vert P_{n,\alpha}^{\beta,c}(h;x)-h(x)\bigr\vert \leq\alpha M \biggl( \frac{ \mu_{n,2}^{\beta,c}(x)}{x} \biggr) ^{r/2}. $$

### Proof

Using Remark 2, we get

3.1 $$ \begin{aligned}[b] \bigl\vert P_{n,\alpha}^{\beta,c} \bigl(h(t);x\bigr)-h(x)\bigr\vert &\leq P_{n,\alpha }^{\beta,c} \bigl( \bigl\vert h(t)-h(x)\bigr\vert ;x \bigr) \\ &\leq\alpha P_{n}^{\beta,c} \bigl( \bigl\vert h(t)-h(x)\bigr\vert ;x \bigr) \\ &\leq\alpha MP_{n}^{\beta,c} \biggl( \frac{\vert t-x\vert ^{r}}{(t+x)^{r/2}};x \biggr) \\ &\leq\frac{\alpha M}{x^{r/2}}P_{n}^{\beta,c} \bigl( \vert t-x \vert^{r};x \bigr). \end{aligned} $$

Taking $p=\frac{2}{r}$ and $q=\frac{2}{2-r}$ and applying Hölder’s inequality, we obtain

3.2 $$ P_{n}^{\beta,c} \bigl( \vert t-x \vert^{r};x \bigr) \leq\bigl\{ P_{n}^{\beta,c} \bigl((t-x)^{2};x\bigr) \bigr\} ^{r/2} \bigl\{ P_{n}^{\beta,c}\bigl(1^{\frac{2}{2-r}};x\bigr) \bigr\} ^{ \frac{2-r}{2}} = \bigl( \mu_{n,2}^{\beta,c}(x) \bigr) ^{r/2}. $$

Combining (3.1) and (3.2), we get

$$\bigl\vert P_{n,\alpha}^{\beta,c}\bigl(h(t);x\bigr)-h(x)\bigr\vert \leq\alpha M \biggl( \frac{ \mu_{n,2}^{\beta,c}(x)}{x} \biggr) ^{r/2}. $$

This completes the proof. □

In the following, we present some weighted approximation results. First, we recall some basic notations. Let $\operatorname {B}_{2}[0,\infty)=\{h:[0, \infty)\to {\mathbb {R}}:\vert h(x)\vert\leq M_{h}(1+x^{2})\mbox{ for all }x\in [0,\infty) \}$. Further, let $\operatorname {C}_{2}[0,\infty)$ be the subspace of $\operatorname {B}_{2}[0,\infty)$ consisting of continuous functions defined on $[0,\infty)$. The norm in $\operatorname {C}_{2}[0,\infty)$ is given by

$$\Vert h\Vert _{2}=\sup_{x\in[0,\infty)}\frac{\vert h(x)\vert}{1+x^{2}}. $$

Also, let

$$\operatorname {C}_{2}^{0}[0,\infty):= \biggl\{ h\in \operatorname {C}_{2}[0,\infty): \lim_{x\to\infty}\frac{\vert h(x)\vert}{1+x^{2}} \text{ is finite} \biggr\} . $$

The next theorem provides us the degree of approximation of $P_{n,\alpha}^{\beta,c}$ in terms of the classical modulus of continuity for the functions in the weighted space $\operatorname {C}_{2}[0, \infty)$.

### Theorem 2

*For* $h\in \operatorname {C}_{2}[0,\infty)$, *we have*

$$\bigl\vert P_{n,\alpha}^{\beta,c}(h;x)-h(x)\bigr\vert \leq4\alpha M_{h}\bigl(1+x ^{2}\bigr)\mu_{n,2}^{\beta,c}(x) + ( 1+\sqrt{\alpha} ) \omega_{b+1} \Bigl( h;\sqrt{ \mu_{n,2}^{\beta,c}(x)} \Bigr), $$

*where* $\omega_{b+1}(h;\delta)$ *is the modulus of continuity of* *h* *on* $[0,b+1]$.

### Proof

From [29], for $x\in[0,b]$ and $t\geq0$, we obtain

$$\bigl\vert h(t)-h(x)\bigr\vert \leq4M_{h}(t-x)^{2} \bigl(1+x^{2}\bigr) + \biggl( 1+\frac{\vert t-x\vert}{ \delta} \biggr) \omega_{b+1}(h;\delta). $$

Applying Remark 2 and the Cauchy–Schwarz inequality, we get

$$\begin{aligned} \bigl\vert P_{n,\alpha}^{\beta,c}(h;x)-h(x)\bigr\vert \leq& 4M_{h}\bigl(1+x ^{2}\bigr)P_{n,\alpha}^{\beta,c} \bigl((t-x)^{2};x\bigr) +\omega_{b+1}(h;\delta) \biggl( 1+ \frac{1}{\delta}\bigl(\alpha\mu_{n,2}^{\beta,c}(x) \bigr)^{1/2} \biggr) \\ \leq& 4\alpha M_{h} \bigl( 1+x^{2} \bigr) \mu_{n,2}^{\beta,c}(x) + \omega_{b+1}(h;\delta) \biggl( 1+ \frac{\sqrt{\alpha}}{\delta}\bigl(\mu_{n,2}^{\beta,c}(x)\bigr)^{1/2} \biggr). \end{aligned}$$

Choosing $\delta=\sqrt{\mu_{n,2}^{\beta,c}(x)}$, we get the desired result. □

To determine the rate of convergence for functions in $\operatorname {C}_{2}^{0}[0, \infty)$, Yüksel and Ispir [6] introduced the weighted modulus of continuity as

$$\Omega(h;\delta)=\sup_{x\in[0,\infty),0< \eta< \delta} \frac{ \vert h(x+\eta)-h(x)\vert}{1+(x+\eta)^{2}}. $$

In the following lemma, we state the properties of the weighted modulus of continuity $\Omega(h;\delta)$.

### Lemma 4

(see [6])

*Let* $h\in \operatorname {C}_{2}^{0}[0,\infty)$. *Then the following results hold*.

1. $\Omega(h;\delta)$ *is monotonically increasing in* *δ*.
2. $\lim_{\delta\to0^{+}}\Omega(h;\delta)=0$.
3. *For each* $m\in {\mathbb {N}}$, $\Omega(h;m\delta)\leq m\Omega(h;\delta)$.
4. *For each* $\lambda\in[0,\infty)$, $\Omega(h;\lambda\delta)\leq(1+ \lambda)\Omega(h;\delta)$.

### Theorem 3

*Let* $h\in \operatorname {C}_{2}^{0}[0,\infty)$, $\beta=\beta(n)\to0$ *as* $n\to\infty$ *with* $\lim_{n\to\infty}n\beta=l\in {\mathbb {R}}$, *and* $b>0$. *Then*

$$\lim_{n\to\infty}\sup_{x\in[0,\infty)} \frac{\vert P_{n,\alpha} ^{\beta,c}(h;x)-h(x)\vert}{(1+x^{2})^{1+b}}=0. $$

### Proof

Let $x_{0}\in[0,\infty)$ be arbitrary but fixed. Then

3.3 $$\begin{aligned}& \sup_{x\in[0,\infty)} \frac{\vert P_{n,\alpha}^{\beta,c}(h;x)-h(x)\vert}{(1+x ^{2})^{1+b}} \\& \quad \leq\sup_{x\leq x_{0}} \frac{\vert P_{n,\alpha}^{ \beta,c}(h;x)-h(x)\vert}{(1+x^{2})^{1+b}} +\sup _{x>x_{0}} \frac{ \vert P_{n,\alpha}^{\beta,c}(h;x)-h(x)\vert}{(1+x^{2})^{1+b}} \\& \quad \leq\bigl\Vert P_{n,\alpha}^{\beta,c}(h;x)-h(x)\bigr\Vert _{\operatorname {C}[0,x _{0}]} +\Vert h\Vert _{2}\sup_{x>x_{0}} \frac{P_{n,\alpha}^{\beta,c}(1+t ^{2};x)}{(1+x^{2})^{1+b}} +\sup_{x>x_{0}} \frac{\vert h(x)\vert }{(1+x^{2})^{1+b}}. \end{aligned}$$

Since $\vert h(x)\vert\leq \Vert h\Vert _{2}(1+x^{2})$, we have

$$\sup_{x>x_{0}} \frac{\vert h(x)\vert}{(1+x^{2})^{1+b}}\leq\frac {\Vert h\Vert _{2}}{(1+x _{0}^{2})^{b}}. $$

Let $\varepsilon >0$ be arbitrary. We choose $x_{0}$ to be so large that

3.4 $$ \frac{\Vert h\Vert _{2}}{(1+x_{0}^{2})^{b}}< \frac {\varepsilon }{6}. $$

For $\varepsilon >0$, there exists $n_{1}\in {\mathbb {N}}$ such that

$$\bigl\vert P_{n,\alpha}^{\beta,c}\bigl(1+t^{2};x\bigr)- \bigl(1+x^{2}\bigr)\bigr\vert < \frac{\varepsilon }{3 \Vert h\Vert _{2}} \quad \text{for all } n\geq n_{1}. $$

Hence, using (3.4), we get

3.5 $$ \begin{aligned}[b] \Vert h\Vert _{2}\sup _{x\geq x_{0}} \frac{P_{n,\alpha}^{\beta,c}(1+t^{2};x)}{(1+x ^{2})^{1+b}} &\leq \Vert h\Vert _{2} \sup_{x\geq x_{0}} \frac{1}{(1+x^{2})^{1+b}} \biggl( \bigl(1+x^{2} \bigr)+\frac{\varepsilon }{3\Vert h\Vert _{2}} \biggr) \\ &\leq \Vert h\Vert _{2}\sup_{x\geq x_{0}} \biggl( \frac{1}{(1+x^{2})^{b}}+\frac{\varepsilon }{3 \Vert h\Vert _{2}(1+x^{2})^{1+b}} \biggr) \\ &\leq \Vert h\Vert _{2}\sup_{x\geq x_{0}} \biggl( \frac{1}{(1+x^{2})^{b}}+\frac{\varepsilon }{3\Vert h\Vert _{2}} \biggr) \\ & \leq\frac{\Vert h\Vert _{2}}{(1+x_{0}^{2})^{b}}+\frac{\varepsilon }{3} \\ &\leq\frac{\varepsilon }{2}. \end{aligned} $$

Applying Theorem 2, we can find $n_{2}\in {\mathbb {N}}$ such that

3.6 $$ \bigl\Vert P_{n,\alpha}^{\beta,c}(h;x)-h(x)\bigr\Vert _{\operatorname {C}[0,x_{0}]} < \frac{\varepsilon }{3}, $$

for all *n* greater than equal to $n_{2}$. Combining (3.3)–(3.6), we obtain

$$\sup_{x\in[0,\infty)} \frac{\vert P_{n,\alpha}^{\beta ,c}(h;x)-h(x)\vert}{(1+x ^{2})^{1+b}}< \varepsilon . $$

This proves the required result. □

In the following theorem, we establish the rate of convergence of the operators $P_{n,\alpha}^{\beta,c}$ in terms of the weighted modulus of continuity Ω.

### Theorem 4

*Let* $h\in \operatorname {C}_{2}^{0}[0,\infty)$. *If* $\beta=\beta(n)\to0$ *as* $n\to\infty$ *and* $\lim_{n\to\infty}n\beta(n)=l\in {\mathbb {R}}$, *then*, *for sufficiently large* *n*, *we have*

$$\sup_{x\in[0,\infty)} \frac{\vert P_{n,\alpha}^{\beta ,c}(h;x)-h(x)\vert}{(1+x ^{2})^{\frac{5}{2}}} \leq C\Omega\biggl( h; \frac{1}{\sqrt{n}} \biggr), $$

*where* *C* *is a positive constant independent of* *h* *and* *n*.

### Proof

For $x\in(0,\infty)$ and $\delta>0$, using the definition of weighted modulus of continuity and Lemma 4, we have

$$\begin{aligned} \bigl\vert h(t)-h(x)\bigr\vert \leq& \bigl(1+\bigl(x+\vert x-t\vert \bigr)^{2}\bigr)\Omega\bigl(h;\vert t-x\vert\bigr) \\ \leq& 2\bigl(1+x^{2}\bigr) \bigl(1+(t-x)^{2}\bigr) \biggl( 1+\frac{\vert t-x\vert}{\delta} \biggr) \Omega(h;\delta). \end{aligned}$$

Applying $P_{n,\alpha}^{\beta,c}(\cdot;x)$ to both sides of the above inequality, we can write

3.7 $$\begin{aligned}& \bigl\vert P_{n,\alpha}^{\beta,c}(h;x)-h(x)\bigr\vert \\& \quad \leq2\bigl(1+x^{2}\bigr)\Omega(h;\delta) \biggl( 1+P_{n,\alpha}^{\beta,c}\bigl((t-x)^{2};x\bigr) +P_{n,\alpha }^{\beta,c} \biggl( \bigl(1+(t-x)^{2}\bigr) \frac{\vert t-x\vert}{\delta };x \biggr) \biggr). \end{aligned}$$

From Lemma 2, for sufficiently large *n*, it follows that

3.8 $$ n\mu_{n,2}^{\beta,c}(x)\leq Cx(cx+2) \quad \text{and}\quad n^{2}\mu_{n,4}^{\beta,c}(x)\leq Cx^{2}(cx+2)^{2}, $$

where *C* is a positive constant. Now, applying the Cauchy–Schwarz inequality in the last term of (3.7), we obtain

3.9 $$\begin{aligned}& P_{n,\alpha}^{\beta,c} \biggl( \bigl( 1+(t-x)^{2} \bigr) \frac{\vert t-x\vert}{ \delta};x \biggr) \\& \quad \leq\frac{1}{\delta} \bigl( \alpha\mu_{n,2}^{ \beta,c}(x) \bigr) ^{1/2} + \frac{1}{\delta} \bigl( \alpha\mu_{n,4} ^{\beta,c}(x) \bigr) ^{1/2} \bigl( \alpha\mu_{n,2}^{\beta,c}(x) \bigr) ^{1/2}. \end{aligned}$$

Combining estimates (3.7)–(3.9) and taking

$$C=2 ( 1+\sqrt{\alpha C}+2\alpha C )\quad \text{and}\quad \delta=\frac{1}{ \sqrt{n}}, $$

we reach the required result. □

Now our aim is to discuss the rate of convergence in terms of the unified Ditzian–Totik modulus of smoothness $\omega_{\phi^{\tau}}(h,t)$, $0\leq\tau\leq1$. First, we define the Ditzian–Totik modulus of smoothness and the Peetre *K*-functional. Let $\phi(x)=\sqrt{x(2+cx)}$ and $h\in \operatorname {C}_{\mathrm{B}}[0,\infty)$. The modulus $\omega_{\phi^{\tau}}(h,t)$, $0\leq\tau\leq1$, is defined as

$$\omega_{\phi^{\tau}}(h,t) =\sup_{0\leq j\leq t} \sup _{x\pm\frac{j\phi^{\tau}(x)}{2}\in[0,\infty)} \biggl\vert h \biggl( x+\frac{j \phi^{\tau}(x)}{2} \biggr) -h \biggl( x-\frac{j\phi^{\tau}(x)}{2} \biggr) \biggr\vert , $$

and the appropriate *K*-functional is given by

$$K_{\phi^{\tau}}(h,t)=\inf_{g\in W_{\tau}} \bigl\{ \Vert h-g\Vert +t \bigl\Vert \phi^{\tau}g'\bigr\Vert \bigr\} , $$

where $W_{\tau}$ is the subspace of the space of locally absolutely continuous functions *g* on $[0,\infty)$, with $\Vert \phi^{\tau }g'\Vert < \infty$. By [30, Theorem 2.1.1], there exists a constant $N>0$ such that

3.10 $$ N^{-1}\omega_{\phi^{\tau}}(h,t) \leq K_{\phi^{\tau}}(h,t)\leq N \omega_{\phi^{\tau}}(h,t). $$

### Theorem 5

*If* $h\in \operatorname {C}_{\mathrm{B}}[0,\infty)$, *then*

$$\bigl\vert P_{n,\alpha}^{\beta,c}(h;x)-h(x)\bigr\vert \leq C \omega_{\phi^{\tau}} \biggl( h,\frac{\phi^{1-\tau}(x)}{\sqrt {n}} \biggr) $$

*for sufficiently large* *n*, *where* *C* *is independent of* *h* *and* *n*.

### Proof

By the definition of $K_{\phi^{\tau}}(h,t)$, there exists a function $g\in W_{\tau}$ such that

3.11 $$ \Vert h-g\Vert +\frac{\phi^{1-\tau}(x)}{\sqrt {n}}\bigl\Vert \phi ^{\tau}g'\bigr\Vert \leq2K _{\phi^{\tau}} \biggl( h, \frac{\phi^{1-\tau}(x)}{\sqrt{n}} \biggr). $$

We can write

3.12 $$ \begin{aligned}[b] \bigl\vert P_{n,\alpha}^{\beta,c}(h;x)-h(x) \bigr\vert &\leq\bigl\vert P_{n, \alpha}^{\beta,c}(h-g;x)\bigr\vert +\bigl\vert P_{n,\alpha}^{\beta,c}(g;x)-g(x)\bigr\vert + \bigl\vert g(x)-h(x)\bigr\vert \\ &\leq2\Vert h-g\Vert +\bigl\vert P_{n,\alpha}^{\beta,c}(g;x)-g(x) \bigr\vert . \end{aligned} $$

Since $g\in W_{\tau}$, we obtain

$$g(t)=g(x)+G(t),\quad \text{where } G(t):= \int_{x}^{t}g'(u)\,\mathrm {d}u, $$

and so

3.13 $$ \bigl\vert P_{n,\alpha}^{\beta,c}(g;x)-g(x)\bigr\vert \leq P_{n,\alpha} ^{\beta,c}\bigl(\vert G\vert;x\bigr). $$

By applying Hölder’s inequality, we get

3.14 $$ \bigl\vert G(t)\bigr\vert \leq\bigl\Vert \phi^{\tau}g'\bigr\Vert \biggl\vert \int_{x}^{t}\frac{\mathrm {d}u}{ \phi^{\tau}(u)}\biggr\vert \leq \bigl\Vert \phi^{\tau}g'\bigr\Vert \vert t-x \vert^{1- \tau} \biggl\vert \int_{x}^{t}\frac{\mathrm {d}u}{\phi(u)}\biggr\vert ^{\tau}. $$

Now

$$\begin{aligned} \biggl\vert \int_{x}^{t}\frac{\mathrm {d}u}{\phi(u)}\biggr\vert \leq& \biggl\vert \int_{x} ^{t}\frac{\mathrm {d}u}{\sqrt{u}}\biggr\vert \biggl( \frac{1}{\sqrt{2+cx}}+\frac{1}{ \sqrt{2+ct}} \biggr) \\ =& 2\vert\sqrt{t}-\sqrt{x}\vert\biggl( \frac{1}{\sqrt{2+cx}}+ \frac{1}{ \sqrt{2+ct}} \biggr) \\ =& \frac{2\vert t-x\vert}{\sqrt{t}+\sqrt{x}} \biggl( \frac {1}{\sqrt{2+cx}}+\frac{1}{ \sqrt{2+ct}} \biggr) \\ \leq& \frac{2\vert t-x\vert}{\sqrt{x}} \biggl( \frac{1}{\sqrt {2+cx}}+\frac{1}{ \sqrt{2+ct}} \biggr), \end{aligned}$$

the inequality $\vert a+b\vert^{\tau}\leq\vert a\vert^{\tau }+\vert b\vert^{\tau}$, $0\leq\tau\leq1$, and (3.14) imply

3.15 $$ \begin{aligned}[b] \bigl\vert G(t)\bigr\vert &\leq \frac{2^{\tau} \Vert \phi^{\tau}g'\Vert \vert t-x\vert}{x^{\tau/2}} \biggl( \frac{1}{\sqrt{2+cx}}+\frac {1}{\sqrt{2+ct}} \biggr) ^{\tau} \\ &\leq\frac{2^{\tau} \Vert \phi^{\tau}g'\Vert \vert t-x\vert}{x^{\tau/2}} \biggl( \frac{1}{(2+cx)^{ \tau/2}}+\frac{1}{(2+ct)^{\tau/2}} \biggr). \end{aligned} $$

Thus, from (3.13), (3.15), and the Cauchy–Schwarz inequality, we get

3.16 $$\begin{aligned}& \bigl\vert P_{n,\alpha}^{\beta,c}(g;x)-g(x) \bigr\vert \\& \quad \leq\frac{2^{\tau} \Vert \phi^{\tau}g'\Vert }{x^{\tau/2}}P_{n,\alpha}^{\beta,c} \biggl( \vert t-x\vert\biggl( \frac{1}{(2+cx)^{\tau/2}} +\frac {1}{(2+ct)^{\tau/2}} \biggr);x \biggr) \\& \quad \leq\frac{2^{\tau} \Vert \phi^{\tau}g'\Vert }{x^{\tau/2}} \biggl( \frac{1}{(2+cx)^{ \tau/2}}\sqrt{\alpha \mu_{n,2}^{\beta,c}} +\sqrt{\alpha\mu_{n,2} ^{\beta,c}} \sqrt{P_{n,\alpha}^{\beta,c} \bigl((2+ct)^{-\tau};x\bigr)} \biggr) \\& \quad \leq 2^{\tau}\bigl\Vert \phi^{\tau}g'\bigr\Vert \sqrt{\alpha\mu_{n,2}^{\beta,c}} \Bigl\{ \phi^{-\tau}(x)+x^{-\tau/2}\sqrt{P _{n,\alpha}^{\beta,c} \bigl( (2+ct)^{-\tau};x \bigr) } \Bigr\} . \end{aligned}$$

Note that for each $x\in(0,\infty)$,

$$P_{n,\alpha}^{\beta,c}\bigl((2+ct)^{-\tau};x\bigr) \to(2+cx)^{-\tau}\quad \text{as } n\to\infty, $$

and thus, for $\varepsilon >0$, there exists $n_{0}\in {\mathbb {N}}$ such that

$$P_{n,\alpha}^{\beta,c} \bigl( (2+ct)^{-\tau};x \bigr) \leq(2+cx)^{- \tau}+\varepsilon \quad \text{for all } n\geq n_{0}. $$

Choosing $\varepsilon =(2+cx)^{-\tau}$, we obtain

$$P_{n,\alpha}^{\beta,c} \bigl( (2+ct)^{-\tau};x \bigr) \leq2(2+cx)^{- \tau} \quad \text{for all } n\geq n_{0}. $$

Therefore, using (3.8) and (3.16), we get

3.17 $$ \begin{aligned}[b] \bigl\vert P_{n,\alpha}^{\beta,c}(g;x)-g(x) \bigr\vert &\leq2^{\tau}\bigl\Vert \phi^{\tau}g' \bigr\Vert \sqrt{\frac{\alpha C\phi^{2}(x)}{n}} \bigl\{ \phi^{- \tau}(x)+ \sqrt{2}x^{-\tau/2}(2+cx)^{-\tau/2} \bigr\} \\ &\leq2^{ \tau}(1+\sqrt{2})\bigl\Vert \phi^{\tau}g' \bigr\Vert \phi^{1-\tau}(x) \sqrt{\frac{ \alpha C}{n}} \end{aligned} $$

for sufficiently large *n*. Thus, from (3.12), (3.17), and (3.11) (in that order), we find

3.18 $$ \begin{aligned}[b] \bigl\vert P_{n,\alpha}^{\beta,c}(h;x)-h(x) \bigr\vert &\leq2\Vert h-g\Vert + 2^{ \tau}(1+\sqrt{2})\bigl\Vert \phi^{\tau}g'\bigr\Vert \phi^{1-\tau}(x)\sqrt{ \frac{ \alpha C}{n}} \\ &\leq C' \biggl\{ \Vert h-g\Vert +\frac{\phi^{1-\tau}(x)}{ \sqrt{n}} \bigl\Vert \phi^{\tau}g'\bigr\Vert \biggr\} \\ &\leq CK_{\phi^{\tau}} \biggl( h,\frac{ \phi^{1-\tau}(x)}{\sqrt{n}} \biggr), \end{aligned} $$

where $C'=\max\{ 2,2^{\tau}(1+\sqrt{2})\sqrt{\alpha C} \} $ and $C=2C'$. By using relation (3.10), we reach the required result. □

Lastly, we obtain the convergence rate for functions having derivatives equivalent with a function of bounded variation. Let $\operatorname {DBV}[0,\infty)$ be the class of functions $h\in \operatorname {B}_{2}[0,\infty)$ having a derivative of bounded variation on every finite subinterval of $[0,\infty)$. The function $h\in \operatorname {DBV}[0,\infty)$ has the representation

$$h(x)= \int_{0}^{x}j(t)\,\mathrm {d}t+h(0), $$

where *j* is a function of bounded variation on each finite subinterval of $[0,\infty)$. For this purpose, we use the following auxiliary result.

### Lemma 5

*For fixed* $u\in(0,\infty)$ *and sufficiently large* *n*, *we have*

3.19 $$ \xi_{n,\alpha}^{\beta,c}(u,v) := \int_{0}^{v}K_{n,\alpha}^{\beta,c}(u;t)\,\mathrm {d}t\leq\alpha\frac{Cu(2+cu)}{n}(u-v)^{-2},\quad 0\leq v< u, $$

*and*

3.20 $$ 1-\xi_{n,\alpha}^{\beta,c}(u,w) = \int_{w}^{\infty}K_{n,\alpha} ^{\beta,c}(u;t)\,\mathrm {d}t\leq\alpha\frac{Cu(2+cu)}{n}(w-u)^{-2},\quad u< w< \infty, $$

*where* *C* *is a positive constant*.

### Proof

Applying Remark 2 and using (3.8), we have

$$\begin{aligned} \xi_{n,\alpha}^{\beta,c}(u,v) =& \int_{0}^{v}K_{n,\alpha}^{\beta,c}(u;t)\,\mathrm {d}t\\ \leq& \int_{0}^{v} \biggl( \frac{u-t}{u-v} \biggr) ^{2} K_{n,\alpha} ^{\beta,c}(u;t)\,\mathrm {d}t\\ =& (u-v)^{-2} \int_{0}^{v}(u-t)^{2}K_{n,\alpha}^{\beta,c}(u;t) \,\mathrm {d}t\\ \leq& \frac{P_{n,\alpha}^{\beta,c} ( (t-u)^{2};u) }{(u-v)^{2}} \leq\frac{\alpha\mu_{n,2}^{\beta,c}(u)}{(u-v)^{2}} \\ \leq& \alpha\frac{C u(2+cu)}{n}(u-v)^{-2}, \end{aligned}$$

showing (3.19). Similarly, applying Remark 2 and using (3.8), we get

$$\begin{aligned} 1-\xi_{n,\alpha}^{\beta,c}(u,w) =& \int_{w}^{\infty}K_{n,\alpha} ^{\beta,c}(u;t)\,\mathrm {d}t\\ \leq& \int_{w}^{\infty} \biggl( \frac{t-u}{w-u} \biggr) ^{2} K_{n, \alpha}^{\beta,c}(u;t)\,\mathrm {d}t\\ =& (w-u)^{-2} \int_{w}^{\infty}(u-t)^{2}K_{n,\alpha}^{\beta,c}(u;t) \,\mathrm {d}t\\ \leq& \frac{P_{n,\alpha}^{\beta,c} ( (u-t)^{2};u) }{(w-u)^{2}} \leq\frac{\alpha\mu_{n,2}^{\beta,c}(u)}{(w-u)^{2}} \\ \leq& \alpha\frac{Cu(2+cu)}{n}(w-u)^{-2}, \end{aligned}$$

showing (3.20). □

### Theorem 6

*Let* $h\in \operatorname {DBV}[0,\infty)$. *Then*, *for every* $x\in(0,\infty)$ *and sufficiently large* *n*, *we have*

$$\begin{aligned} \bigl\vert P_{n,\alpha}^{\beta,c}(h;x)-h(x)\bigr\vert \leq& \frac{1}{ \alpha+1}\bigl\vert h'(x+)+\alpha h'(x-)\bigr\vert \sqrt{ \frac{\alpha Cx(2+cx)}{n}} \\ & {}+\frac{\alpha}{\alpha+1}\bigl\vert h'(x+)-h'(x-)\bigr\vert \sqrt{\frac{ \alpha Cx(2+cx)}{n}} \\ &{}+\frac{\alpha C(2+cx)}{n} \sum _{k=1}^{[\sqrt{n}]} \Biggl( \bigvee _{x-\frac{x}{k}}^{x+\frac{x}{k}}h'_{x} \Biggr) \\ &{} +\frac{x}{\sqrt{n}} \Biggl( \bigvee_{x-\frac{x}{\sqrt {n}}}^{x+\frac{x}{ \sqrt{n}}}h'_{x} \Biggr) +\frac{\alpha C(2+cx)}{nx}\bigl\vert h(2x)-h(x)-xh'(x+)\bigr\vert \\ & {}+\bigl\vert h'(x+)\bigr\vert \sqrt{\frac{\alpha Cx(2+cx)}{n}} \\ &{} + \biggl( 4M+\frac{M+\vert h(x)\vert}{x ^{2}} \biggr) \frac{\alpha Cx(2+cx)}{n}, \end{aligned}$$

*where* $\bigvee_{a}^{b}h(x) $ *represents the total variation of* *h* *on* $[a,b]$, *M* *is a constant*, *and* $h'_{x}$ *is defined by*

3.21 $$ h_{x}'(u)= \textstyle\begin{cases} h'(u)-h'(x-), & \textit{if } 0\leq u< x, \\ 0, & \textit{if } u=x, \\ h'(u)-h'(x+), & \textit{if } x< u< \infty. \end{cases} $$

### Proof

For any $h\in \operatorname {DBV}[0,\infty)$, from (3.21), we may write

3.22 $$\begin{aligned} h'(u) = & h'_{x}(u)+\frac{1}{\alpha+1} \bigl( h'(x+)+ \alpha h'(x-) \bigr) \\ &{}+\frac{1}{2} \bigl( h'(x+)-h'(x-) \bigr) \biggl( \operatorname {sgn}(u-x)+\frac{\alpha -1}{\alpha+1} \biggr) \\ &{}+ \delta_{x}(u) \biggl[ h'(u)-\frac{1}{2} \bigl( h'(x+)+h'(x-) \bigr) \biggr] , \end{aligned}$$

where

$$\delta_{x}(t)= \textstyle\begin{cases} 1, & \text{if } t=x, \\ 0, & \text{if } t\neq x. \end{cases} $$

Since $P_{n,\alpha}^{\beta,c}(1;x)=1$, using (1.4), for every $x\in(0,\infty)$, we get

3.23 $$ \begin{aligned}[b] P_{n,\alpha}^{\beta,c}(h;x)-h(x) &= \int_{0}^{\infty}K_{n,\alpha} ^{\beta,c}(x;t) \bigl(h(t)-h(x)\bigr)\,\mathrm {d}t\\ &= \int_{0}^{\infty}K_{n,\alpha} ^{\beta,c}(x;t) \biggl( \int_{x}^{t}h'(u)\,\mathrm {d}u\biggr) \,\mathrm {d}t. \end{aligned} $$

From (3.22) and (3.23), we get

$$\begin{aligned}& P_{n,\alpha}^{\beta,c}(h;x)-h(x) \\& \quad = \int_{0}^{\infty}K_{n,\alpha }^{\beta,c}(x;t) \int_{x}^{t} \biggl[ h'_{x}(u)+ \frac{1}{\alpha+1} \bigl( h'(x+)+\alpha h'(x-) \bigr) + \frac{1}{2} \bigl( h'(x+)-h'(x-) \bigr) \\& \qquad {}\times \biggl( \operatorname {sgn}(u-x)+\frac{\alpha-1}{\alpha+1} \biggr) +\delta _{x}(u) \biggl[ h'(u)-\frac{1}{2} \bigl( h'(x+)+h'(x-) \bigr) \biggr] \,\mathrm {d}u\biggr] \,\mathrm {d}t\\& \quad = C_{1}+C_{2}+C_{3}+C_{n,\alpha}^{\beta,c} \bigl(h'_{x},x\bigr) +D_{n,\alpha }^{\beta,c} \bigl(h'_{x},x\bigr), \end{aligned}$$

where

$$\begin{aligned}& C_{1}= \int_{0}^{\infty} \biggl( \int_{x}^{t}\frac{1}{\alpha+1} \bigl( h'(x+)+ \alpha h'(x-) \bigr) \,\mathrm {d}u\biggr) K_{n,\alpha}^{\beta,c}(x;t)\,\mathrm {d}t, \\& C_{2}= \int_{0}^{\infty}K_{n,\alpha}^{\beta,c}(x;t) \biggl( \int_{x} ^{t}\frac{1}{2} \bigl( h'(x+)-h'(x-) \bigr) \biggl( \operatorname {sgn}(u-x)+ \frac{ \alpha-1}{\alpha+1} \biggr) \,\mathrm {d}u\biggr) \,\mathrm {d}t, \\& C_{3}= \int_{0}^{\infty} \biggl( \int_{x}^{t} \biggl( h'(u)- \frac{1}{2} \bigl( h'(x+)+h'(x-) \bigr) \biggr) \delta_{x}(u)\,\mathrm {d}u\biggr) K_{n,\alpha }^{\beta,c}(x;t)\,\mathrm {d}t, \\& C_{n,\alpha}^{\beta,c}\bigl(h'_{x},x\bigr)= \int_{0}^{x} \biggl( \int_{x}^{t}h'_{x}(u)\,\mathrm {d}u\biggr) K _{n,\alpha}^{\beta,c}(x;t)\,\mathrm {d}t, \end{aligned}$$

and

$$\begin{aligned}& D_{n,\alpha}^{\beta,c}\bigl(h'_{x},x\bigr)= \int_{x}^{\infty} \biggl( \int_{x} ^{t}h'_{x}(u)\,\mathrm {d}u\biggr) K_{n,\alpha}^{\beta,c}(x;t)\,\mathrm {d}t. \end{aligned}$$

Obviously,

3.24 $$ C_{3}= \int_{0}^{\infty} \biggl( \int_{x}^{t} \biggl( h'(u)- \frac{1}{2} \bigl( h'(x+)+h'(x-) \bigr) \biggr) \delta_{x}(u)\,\mathrm {d}u\biggr) K_{n,\alpha }^{\beta,c}(x;t)\,\mathrm {d}t=0. $$

Next, using (1.4), we get

3.25 $$ \begin{aligned}[b] C_{1} &= \int_{0}^{\infty} \biggl( \int_{x}^{t}\frac{1}{\alpha+1} \bigl( h'(x+)+\alpha h'(x-) \bigr) \,\mathrm {d}u\biggr) K_{n,\alpha}^{\beta,c}(x;t)\,\mathrm {d}t\\ &= \frac{1}{\alpha+1} \bigl( h'(x+)+\alpha h'(x-) \bigr) \int_{0} ^{\infty}(t-x)K_{n,\alpha}^{\beta,c}(x;t) \,\mathrm {d}t\\ &= \frac{1}{\alpha +1} \bigl( h'(x+)+\alpha h'(x-) \bigr) P_{n,\alpha}^{\beta,c} \bigl( (t-x);x \bigr) \end{aligned} $$

and

3.26 $$\begin{aligned} C_{2} = & \int_{0}^{\infty}K_{n,\alpha}^{\beta,c}(x;t) \biggl( \int_{x}^{t}\frac{1}{2} \bigl( h'(x+)-h'(x-) \bigr) \biggl( \operatorname {sgn}(u-x)+ \frac{ \alpha-1}{\alpha+1} \biggr) \,\mathrm {d}u\biggr) \,\mathrm {d}t \\ =& \frac{1}{2} \bigl( h'(x+)-h'(x-) \bigr) \biggl[ - \int_{0}^{x} \biggl( \int_{t}^{x} \biggl( \operatorname {sgn}(u-x)+\frac{\alpha-1}{\alpha+1} \biggr) \,\mathrm {d}u\biggr) K_{n,\alpha}^{\beta,c}(x;t)\,\mathrm {d}t \\ &{}+ \int_{x}^{\infty } \biggl( \int_{x}^{t} \biggl( \operatorname {sgn}(u-x)+\frac{\alpha-1}{\alpha+1} \biggr) \,\mathrm {d}u\biggr) K_{n, \alpha}^{\beta,c}(x;t)\,\mathrm {d}t\biggr] \\ \leq& \frac{\alpha}{\alpha+1}\bigl\vert h'(x+)-h'(x-) \bigr\vert \int_{0}^{\infty }\vert t-x\vert K_{n,\alpha}^{\beta,c}(x;t) \,\mathrm {d}t \\ =& \frac{\alpha}{\alpha+1}\bigl\vert h'(x+)-h'(x-)\bigr\vert P_{n,\alpha}^{ \beta,c} \bigl( \vert t-x\vert;x \bigr). \end{aligned}$$

Combining (3.23)–(3.26), applying Remark 2 and the Cauchy–Schwarz inequality, and using (3.8), we obtain

3.27 $$\begin{aligned}& \bigl\vert P_{n,\alpha}^{\beta,c}(h;x)-h(x) \bigr\vert \\& \quad \leq \frac{1}{ \alpha+1}\bigl\vert h'(x+)+\alpha h'(x-)\bigr\vert \bigl( \alpha P_{n}^{ \beta,c} \bigl( (t-x)^{2};x \bigr) \bigr) ^{1/2} +\frac{\alpha}{\alpha +1} \bigl\vert h'(x+)-h'(x-)\bigr\vert \\& \qquad {}\times \bigl( \alpha P_{n}^{\beta,c} \bigl( (t-x)^{2};x \bigr) \bigr) ^{1/2} +\bigl\vert C_{n,\alpha}^{\beta,c} \bigl(h'_{x},x\bigr)\bigr\vert +\bigl\vert D_{n, \alpha}^{\beta,c}\bigl(h'_{x},x\bigr)\bigr\vert \\& \quad \leq \frac{1}{\alpha+1} \bigl\vert h'(x+)+\alpha h'(x-)\bigr\vert \sqrt{\frac{\alpha Cx(2+cx)}{n}}+\frac{ \alpha}{\alpha+1} \bigl\vert h'(x+)-h'(x-)\bigr\vert \\& \qquad {}\times \sqrt{\frac{ \alpha Cx(2+cx)}{n}}+\bigl\vert C_{n,\alpha}^{\beta,c} \bigl( h'_{x},x \bigr) \bigr\vert + \bigl\vert D_{n,\alpha}^{\beta,c}\bigl(h'_{x},x\bigr)\bigr\vert . \end{aligned}$$

Now we estimate $C_{n,\alpha}^{\beta,c}(h'_{x},x)$ and $D_{n,\alpha }^{\beta,c}(h'_{x},x)$. Since

$$\int_{a}^{b}\frac{\mathrm{d}}{\mathrm {d}t} \xi_{n,\alpha}^{\beta,c}(x,t)\,\mathrm {d}t\leq1 \quad \text{for all } [a,b]\subseteq[0, \infty), $$

substituting $y=x-x/\sqrt{n}$ and applying Lemma 5, we get

$$\begin{aligned} \bigl\vert C_{n,\alpha}^{\beta,c}\bigl(h'_{x},x \bigr)\bigr\vert =& \biggl\vert \int_{0} ^{x} \biggl( \int_{x}^{t}h'_{x}(u)\,\mathrm {d}u\biggr) {\,\mathrm{d}}_{t}\xi_{n,\alpha} ^{\beta,c}(x,t)\biggr\vert = \biggl\vert \int_{0}^{x}\xi_{n,\alpha}^{\beta,c}(x,t)h'_{x}(t) \,\mathrm {d}t\biggr\vert \\ \leq& \int_{0}^{y}\bigl\vert h'_{x}(t) \bigr\vert \bigl\vert \xi_{n,\alpha }^{\beta,c}(x,t)\bigr\vert \,\mathrm {d}t+ \int_{y}^{x}\bigl\vert h'_{x}(t) \bigr\vert \bigl\vert \xi_{n,\alpha}^{\beta,c}(x,t)\bigr\vert \,\mathrm {d}t\\ \leq& \frac{\alpha Cx(2+cx)}{n} \int_{0}^{y} \Biggl( \bigvee _{t}^{x}h'_{x} \Biggr) (x-t)^{-2}\,\mathrm {d}t+ \int_{y}^{x} \Biggl( \bigvee _{t}^{x}h'_{x} \Biggr) \,\mathrm {d}t\\ \leq& \frac{\alpha Cx(2+cx)}{n} \int_{0}^{y} \Biggl( \bigvee _{t}^{x}h'_{x} \Biggr) (x-t)^{-2}\,\mathrm {d}t+\frac{x}{\sqrt{n}} \Biggl( \bigvee _{x-\frac{x}{\sqrt{n}}} ^{x}h'_{x} \Biggr) \\ =& \frac{\alpha Cx(2+cx)}{n} \int_{0}^{x-\frac{x}{\sqrt{n}}} \Biggl( \bigvee _{t}^{x}h'_{x} \Biggr) (x-t)^{-2}\,\mathrm {d}t+\frac{x}{\sqrt{n}} \Biggl( \bigvee _{x-\frac{x}{\sqrt {n}}}^{x}h'_{x} \Biggr). \end{aligned}$$

Substituting $u=x/(x-t)$, we obtain

$$\begin{aligned} \frac{\alpha Cx(2+cx)}{n} \int_{0}^{x-\frac{x}{\sqrt{n}}}(x-t)^{-2} \Biggl( \bigvee _{t}^{x}h'_{x} \Biggr) \,\mathrm {d}t =& \frac{\alpha Cx(2+cx)}{n}x^{-1} \int_{1}^{\sqrt{n}} \Biggl( \bigvee _{x-\frac{x}{u}}^{x}h'_{x} \Biggr) \,\mathrm {d}u\\ \leq& \frac{\alpha C(2+cx)}{n} \sum_{k=1}^{[\sqrt{n}]} \Biggl( \bigvee_{x- \frac{x}{k}}^{x}h'_{x} \Biggr). \end{aligned}$$

Thus,

3.28 $$ \bigl\vert C_{n,\alpha}^{\beta,c}\bigl(h'_{x},x \bigr)\bigr\vert \leq\frac{\alpha C(2+cx)}{n} \sum_{k=1}^{[\sqrt{n}]} \Biggl( \bigvee_{x- \frac{x}{k}}^{x}h'_{x} \Biggr) +\frac{x}{\sqrt{n}} \Biggl( \bigvee_{x-\frac{x}{ \sqrt{n}}}^{x}h'_{x} \Biggr). $$

Again, using the Cauchy–Schwarz inequality, integration by parts, and applying Lemma 5 to estimate $D_{n,\alpha}^{\beta,c}(h'_{x},x)$, we get

$$\begin{aligned} \bigl\vert D_{n,\alpha}^{\beta,c}\bigl(h'_{x},x \bigr)\bigr\vert \leq& \biggl\vert \int_{2x}^{\infty} \biggl( \int_{x}^{t}h'_{x}(u)\,\mathrm {d}u\biggr) K_{n,\alpha} ^{\beta,c}(x;t)\,\mathrm {d}t\biggr\vert \\ &{}+\biggl\vert \int_{x}^{2x} \biggl( \int_{x}^{t}h'_{x}(u)\,\mathrm {d}u\biggr) {\,\mathrm{d}} _{t} \bigl( 1-\xi_{n,\alpha}^{\beta,c}(x,t) \bigr) \biggr\vert \\ \leq& \biggl\vert \int_{2x}^{\infty}h(t)K_{n,\alpha}^{\beta,c}(x;t) \,\mathrm {d}t\biggr\vert +\bigl\vert h(x)\bigr\vert \biggl\vert \int_{2x}^{\infty}K_{n,\alpha}^{\beta,c}(x;t)\,\mathrm {d}t\biggr\vert \\ & {}+\bigl\vert h'(x+)\bigr\vert \biggl( \int_{2x}^{\infty}(t-x)^{2} K_{n,\alpha}^{\beta,c}(x;t)\,\mathrm {d}t\biggr) ^{1/2} \\ & {}+\frac{\alpha C(2+cx)}{nx} \biggl\vert \int_{x}^{2x} \bigl( h'(u)-h'(x+) \bigr) \,\mathrm {d}u\biggr\vert +\biggl\vert \int_{x}^{x+\frac{x}{\sqrt{n}}}h'_{x}(t)\,\mathrm {d}t\biggr\vert \\ &{} +\frac{\alpha Cx(2+cx)}{n} \biggl\vert \int_{x+\frac{x}{\sqrt{n}}}^{2x}(t-x)^{-2}h'_{x}(t) \,\mathrm {d}t\biggr\vert . \end{aligned}$$

$D_{n,\alpha}^{\beta,c}(h'_{x},x)$ is estimated in a manner similar to $C_{n,\alpha}^{\beta,c}(h'_{x},x)$. Putting $t=x+x/u$ and using (3.8), we get

3.29 $$\begin{aligned} \bigl\vert D_{n,\alpha}^{\beta,c} \bigl(h'_{x},x\bigr)\bigr\vert \leq& M \int_{2x}^{ \infty}\bigl(1+t^{2} \bigr)K_{n,\alpha}^{\beta,c}(x;t)\,\mathrm {d}t \\ &{}+\bigl\vert h(x)\bigr\vert \int_{2x}^{\infty}K_{n,\alpha}^{\beta,c}(x;t)\,\mathrm {d}t+\bigl\vert h'(x+)\bigr\vert \sqrt{\frac{\alpha Cx(2+cx)}{n}} \\ &{}+ \frac{\alpha C(2+cx)}{nx}\bigl\vert h(2x)-h(x)-xh'(x+)\bigr\vert + \frac{x}{\sqrt{n}} \Biggl( \bigvee_{x}^{x+\frac{x}{\sqrt{n}}}h'_{x} \Biggr) \\ &{}+ \frac{ \alpha C(2+cx)}{n} \biggl\vert \int_{x+\frac{x}{\sqrt{n}}}^{2x}(t-x)^{-2}h'_{x}(t) \,\mathrm {d}t\biggr\vert \\ \leq& M \int_{2x}^{\infty}\bigl(1+t^{2} \bigr)K_{n,\alpha}^{\beta,c}(x;t)\,\mathrm {d}t+\bigl\vert h(x)\bigr\vert \int_{2x}^{\infty}K_{n,\alpha}^{\beta,c}(x;t)\,\mathrm {d}t \\ &{} +\bigl\vert h'(x+)\bigr\vert \sqrt{\frac{\alpha Cx(2+cx)}{n}} \\ &{}+ \frac{\alpha C(2+cx)}{nx}\bigl\vert h(2x)-h(x)-xh'(x+)\bigr\vert + \frac{x}{\sqrt{n}} \Biggl( \bigvee_{x}^{x+\frac{x}{\sqrt{n}}}h'_{x} \Biggr) \\ &{}+ \frac{ \alpha C(2+cx)}{n} \sum_{k=1}^{[\sqrt{n}]} \Biggl( \bigvee_{x}^{x+\frac{x}{ \sqrt{n}}}h'_{x} \Biggr). \end{aligned}$$

For $t\geq2x$, we have $t\leq2(t-x)$ and $x\leq t-x$. Now, using (3.8), we obtain

$$\begin{aligned}& M \int_{2x}^{\infty}t^{2}K_{n,\alpha}^{\beta,c}(x;t) \,\mathrm {d}t+ \bigl( M+\bigl\vert h(x)\bigr\vert \bigr) \int_{2x}^{\infty }K_{n,\alpha}^{\beta,c}(x;t)\,\mathrm {d}t\\& \quad \leq 4M \int_{2x}^{\infty}(t-x)^{2}K_{n,\alpha}^{\beta,c}(x;t) \,\mathrm {d}t+\bigl(M+\bigl\vert h(x)\bigr\vert \bigr) \int_{2x}^{\infty}\frac{(t-x)^{2}}{x^{2}} K_{n, \alpha}^{\beta,c}(x;t) \,\mathrm {d}t\\& \quad \leq 4M \int_{0}^{\infty}(t-x)^{2}K_{n,\alpha}^{\beta,c}(x;t) \,\mathrm {d}t+\frac{(M+\vert h(x)\vert)}{x^{2}} \int_{0}^{\infty}(t-x)^{2}K_{n, \alpha}^{\beta,c}(x;t) \,\mathrm {d}t\\& \quad = \biggl( 4M+\frac{M+\vert h(x)\vert}{x^{2}} \biggr) \frac {\alpha Cx(2+c x)}{n}. \end{aligned}$$

Combining this with (3.27)–(3.29) yields the desired result. □

## Conclusion

The Bézier variant of a sequence of mixed hybrid operators has been introduced and the rate of convergence by means of the Lipschitz class and the modulus of continuity has been established. The weighted approximation properties and a direct approximation theorem have been obtained. The approximation of functions with derivatives of bounded variation has been studied.
